# A Case of Ruptured Aortic Arch Aneurysm Successfully Treated by Thoracic Endovascular Aneurysm Repair with Chimney Graft

**DOI:** 10.1155/2015/780147

**Published:** 2015-02-28

**Authors:** Yohei Kawatani, Yujiro Hayashi, Yujiro Ito, Hirotsugu Kurobe, Yoshitsugu Nakamura, Yuji Suda, Takaki Hori

**Affiliations:** Department of Cardiovascular Surgery, Chiba-Nishi General Hospital, 107-1 Kanegasaku Matsudo-shi, Chiba-ken 2702251, Japan

## Abstract

We report the case of aortic arch aneurysm rupture treated successfully with thoracic endovascular aneurysm repair (TEVAR) accompanied by aortic arch debranching using the chimney graft technique. A 94-year-old man was transported to the hospital after complaining of chest pain for one day. Contrast-enhanced computed tomographic (CT) images revealed an aortic arch aneurysm rupture. Considering the patient's age and postoperative activities of daily living, TEVAR was used. In order to place an indwelling stent graft from the ascending aorta to the periphery, the chimney graft technique was used to debranch the brachiocephalic artery. Hemodynamics was stabilized postsurgically. Plain CT performed 20 days postoperatively confirmed that the intrathoracic hematoma had decreased in size. Although respiratory failure was persistent, there were improvements and the patient was extubated 34 days postoperatively and discharged from the intensive care unit 37 days postoperatively. On postoperative day 75, he was discharged from the hospital to an elder care facility. Few reports have focused on stent grafting for treating aortic arch aneurysm rupture. TEVAR using the chimney graft technique could be an effective treatment option for patients with a decreased ability to tolerate surgery.

## 1. Introduction

Thoracic aortic rupture is a condition with a high fatality rate. The survival rate is low in the absence of surgical treatment; thus, early diagnosis and early therapeutic intervention are critically important. Commonly, the surgical procedure consists of artificial blood vessel replacement under thoracotomy. Artificial blood vessel replacement surgery is a highly invasive surgical procedure, and despite improvements in anesthetic management, surgical procedures, and perioperative management, the condition continues to have a high mortality rate and incidence of complications [1]. In contrast, a number of studies have recently reported the use of stent grafting, a minimally invasive surgical procedure, for the treatment of thoracic aortic rupture.

Here we report, along with a discussion of the literature, our experience with a case of aortic arch aneurysm rupture in which the patient survived as a result of stent grafting accompanied by aortic arch debranching using the chimney graft technique.

## 2. Case Presentation

The patient was a 94-year-old man presented with chest and back pain, which had begun to develop the day before his admission to the hospital. The patient initially stayed at home in bed to rest; however, the symptoms increased rapidly and he requested to be transported to the emergency department. On admission, his heart rate was 80 beats per minute and his blood pressure was 117/67 mmHg, which fell to uncountable levels at the emergency room during examination. Oxygen saturation was 97%. His level of consciousness based on the Glasgow Coma Scale was E 2, V 4, M 6. He showed slight confusion but was able to communicate and interact. A complete blood count and serum chemistry panel revealed the following: leukocyte count, 7090/*μ*L; red blood cell count, 286 × 10^4^/*μ*L; hemoglobin, 9.3 g/dL; platelets, 13.4 × 10^4^/*μ*L; aspartate aminotransferase, 15 IU/L; alanine aminotransferase, 8 IU/L; and alkaline phosphatase, 227 IU/L. Additional blood work revealed the following: *γ*-glutamyl transpeptidase, 13 IU/L; C-reactive protein, 6.26 mg/dL; prothrombin time-international normalized ratio, 1.16; activated partial thromboplastin time, 22.6 sec; fibrinogen, 308 mg/dL; and D-dimer, 27.1 *μ*g/mL.

**Figure 1:**
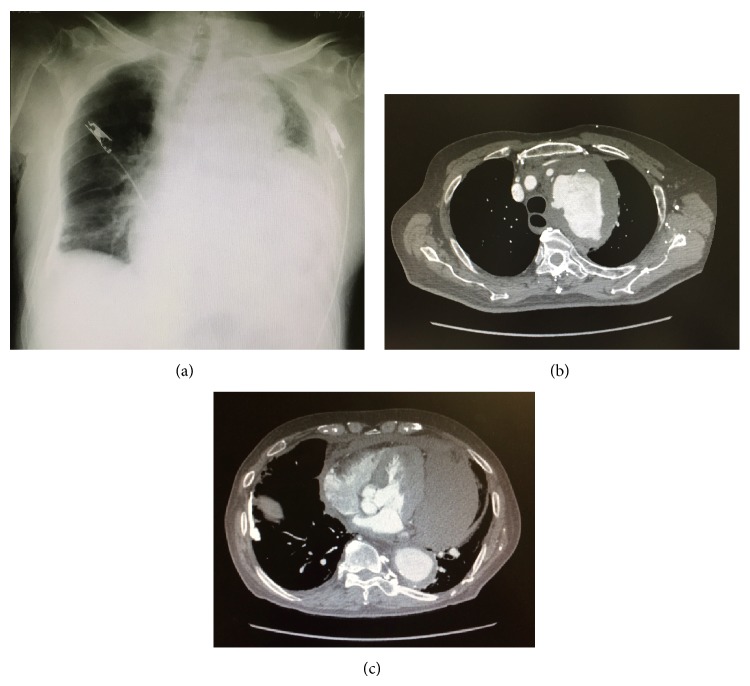
(a) A plain radiograph of the patient's chest revealed a massive left pleural effusion. (b) and (c) Contrast-enhanced computed tomography revealed the presence of an aortic arch aneurysm surrounded by a hematoma, as well as extravasation of contrast media. The hematoma spread from around the aorta towards the mediastinum.

A plain radiograph of the chest revealed decreased transparency of the left thoracic cavity and expanded mediastinal shadow. Contrast-enhanced computed tomography revealed an aortic arch aneurysm and extravasation of the contrast agent from the area surrounding the aneurysm into the mediastinum and thoracic cavity (Figure 1). The condition was diagnosed as a rupture of a thoracic aortic aneurysm. In consideration of the patient's age and his ability to tolerate surgery, a therapeutic strategy using stent grafting (thoracic endovascular aortic repair, TEVAR) was adopted. Since the aneurysm was located on the aortic arch, arrangements were made to achieve a landing of the proximal end on the ascending aorta. For that reason, a debranching of the brachiocephalic artery was needed. We therefore decided to conduct the procedure using the chimney graft technique.

During surgery, the right common carotid artery was exposed, and an indwelling 9-Fr sheath was put in place. A chimney graft was constructed by introducing the guide wire into the ascending aorta and placing a stent graft (*Excluder*, 16 × 95 mm, W. L. Gore & Associates, Newark, DE), which was introduced from the brachiocephalic artery into the ascending aorta. Next, a bypass was created between the right common carotid artery and the left common carotid artery by using an artificial blood vessel (*Fusion*, 8 mm; Maquet GmbH & Co., KG, Mannheim, Germany). The left and right common carotid artery were exposed, and the artificial blood vessel was placed and passed though subcutaneously. The right common carotid artery was incised at the placement site of the indwelling sheath and was anastomosed with the artificial blood vessel. The left common carotid artery was blocked and was detached. An end-to-end anastomosis was created between the distal stump and the artificial blood vessel, and the proximal stump was ligated.

**Figure 2:**
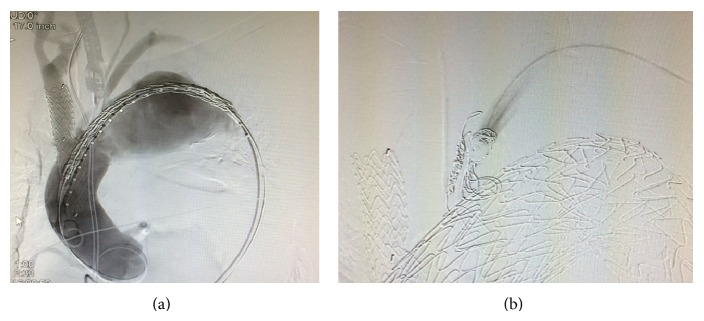
Contrast images taken during surgery. (a) After an indwelling chimney graft was placed from the brachiocephalic artery into the ascending aorta, an indwelling C-TAG was placed from the ascending aorta into the distal aortic arch. (b) After an indwelling C-TAG was placed, the left subclavian artery was coil-embolized. The findings confirmed that endoleaks from the left subclavian artery were fully controlled.

The common femoral artery was exposed on both sides. Two indwelling stent grafts (*Conformable GORE TAG* 45 × 150 mm and 37 × 150 mm, W. L. Gore & Associates, Newark, DE) were placed using the femoral artery approach from the ascending aorta into the aortic arch. To prevent endoleaks from the left subclavian artery, the latter was coil-embolized at its origin. Because the surgery was emergency operation to save the patient's life, we did not perform axilloaxillar bypass even though we excluded left subclavian artery in the operation (Figure 2). The patient returned to the ICU once; an impairment of blood return was found in the left upper extremity, and a bypass was created between the right and left axillary arteries using an artificial blood vessel (*Fusion*, 8 mm, Maquet GmbH & Co., KG, Mannheim, Germany).

### 2.1. Postoperative Course

**Figure 3:**
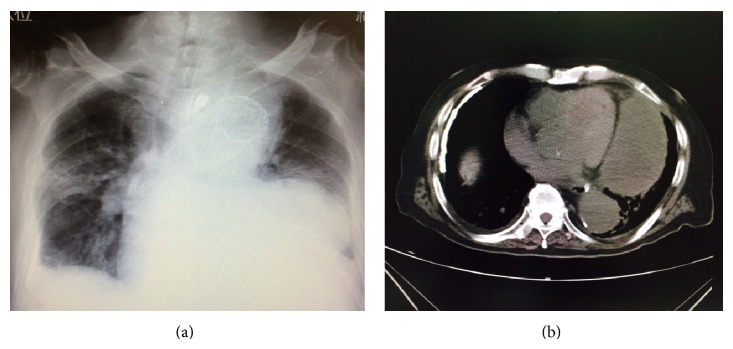
(a) Plain radiograph taken at postoperative day 25. (b) Image from plain CT performed at postoperative day 20. Resorption of the hematoma had progressed, and its size had decreased. The findings revealed that hemostasis was achieved as a result of TEVAR and that the patient's condition tended to improve.

The patient's condition required blood transfusion and fluid replacement; however, hemodynamics remained stable, and he recovered from the state of shock. Evaluations following the end of sedation showed no neurological abnormalities, such as motor paralysis or disturbance of consciousness. After extubation, the patient was capable of holding a conversation and responding properly. There were no findings to indicate neurological complications, such as paralysis of the extremities. The patient's respiratory status began to deteriorate immediately after surgery; withdrawal of mechanical ventilation was impossible for a long period of time. The patient was finally extubated 34 days postoperatively and was discharged from the ICU 37 days postoperatively (Figure 3). On postoperative day 75, he was transferred to another hospital for rehabilitation.

## 3. Discussion

As noted previously, rupture of a thoracic aortic aneurysm is a condition with a high mortality rate, and for the patient to survive, the bleeding must be controlled as early as possible through surgical treatment. In general, surgical treatment consists of artificial blood vessel replacement performed under thoracotomy. Despite improvements in surgical techniques, extracorporeal circulation, and methods for systemic management during anesthesia, the survival rate after surgical treatment has been reported to be as low as 24.7% [1].

In patients with a decreased ability to tolerate surgery, such as the elderly, surgery is often difficult to perform because of its invasiveness or because of a poor preoperative condition caused by the severity of the disease itself. In addition, the fatality rate and the incidence of complications are extremely high, even in patients who receive surgical treatment. Furthermore, even if the patient survives, the patient's ability to perform activities of daily living decreases considerably after surgery.

Conversely, compared to artificial blood vessel replacement performed under thoracotomy, stent grafting is a minimally invasive surgery because it does not require extracorporeal circulation or hypothermic circulatory arrest. In recent years, cases using stent grafting to treat aortic aneurysm ruptures have been reported. However, there have been few reports on the use of stent grafting to treat aortic arch aneurysm ruptures requiring branch reconstruction [2]. A review of 100 reports on thoracic aortic aneurysm ruptures, including 76 cases using TEVAR and 24 cases using surgical repair under thoracotomy (descending thoracic aneurysm repair, DTAR), showed that the mortality rate associated with TEVAR was 8% and that associated with DTAR was 29%. It also showed that the short-term outcomes of TEVAR were better than those of traditional open repair. In addition, the duration of hospitalization tended to be shorter in the TEVAR group, and although there was no significant difference between the two groups in terms of the incidence of postoperative complications, such as cerebral infarction, myocardial infarction, acute renal impairment, and paraplegia, the incidence of respiratory complications was significantly lower with TEVAR [3].

When stent grafting is used for the treatment of aortic arch aneurysms, a debranching of the cervical branches must be performed because of the positional relationship between the cervical branches and the aneurysm requiring treatment. Because the aneurysm in the present case was located at the top of the aortic arch, the indwelling stent graft needed to be introduced from the ascending aorta into the descending aorta in order for the stent graft to achieve full therapeutic effect. In such cases, the brachiocephalic artery, the left common carotid artery, and the left subclavian artery must all be debranched. A number of techniques can be used for debranching the brachiocephalic artery. One technique consists of thoracotomy followed by debranching of the brachiocephalic artery by disconnecting the latter at its origin and anastomosing it to the ascending aorta [3]. Another technique is the chimney graft technique, which uses an approach from the common carotid artery and in which the stent graft is introduced from the ascending aorta into the brachiocephalic artery. The chimney graft technique allows for an intravascular approach and does not require thoracotomy; therefore, it reduces respiratory complications, and, in that regard, is less invasive compared to the technique combining debranching and thoracotomy. In addition, the chimney graft technique has also been reported to be associated with a low mortality [4]. There have been some concerns raised in terms of the frequency of complication with cerebral infarction following the use of the chimney graft technique, but the incidence of complications varied depending on the report. According to a review published by Hogendoorn et al., there have been a total of 101 reports on the use of chimney grafts in 94 patients, and the analysis of these cases revealed the presence of cerebral infarction in 18% of the cases. In addition, the mortality rate at day 30 was 3.2%; all deaths were due to procedure-related stroke [5]. Thus, the chimney graft technique is a procedure that poses concerns due to the high frequency of cerebral infarction as a complication; however, for cases in which surgical treatment using thoracotomy seems unfeasible, surgery cannot be performed unless the chimney graft technique is used. In other words, in some cases, using the chimney graft makes surgery possible, allowing for the patient's survival. In this regard, using the chimney graft technique may be meaningful.

In the present case, the chimney graft technique was chosen by taking into account the complications associated with chimney grafts and the degree of invasiveness of other methods. The intraoperative findings in the present case were as follows. An incision was made in the right common carotid artery in order to create a right-left common carotid artery bypass. The lumen was put under observation, and as a result, an extensive dissociation of the intima was found at the portion where a purse string suture had been placed in order to achieve hemostasis after sheath removal. The dissociation of the intima was repaired, and an anastomosis with the artificial blood vessel was created. Our hospital has experienced cases in which the placing of an indwelling chimney graft was complicated by extensive cerebral ischemia. Similar complications have also been reported in a number of reports published from other institutions. One possible cause may be the dissociation of the intima at the area from which the sheath had been removed. Repairing the dissociated intima may have helped avoid any complications of cerebral infarction due to the chimney graft.

The intraoperative findings in this case, as well as our experience with vessel wall repair, suggest that, in order to reduce complications following chimney graft placement, angiotomy followed by angioplasty should be performed instead of placing a purse string suture for hemostasis, even when there is no need to make an incision to create an anastomosis, as in the present case. In addition, various devices are currently being used for the chimney graft technique; however, the majority consists of devices used in the percutaneous transluminal angioplasty of the iliac artery [4]. If devices for use in cervical branches and graft edges causing less damage to the intima could be developed, further reductions in complications, such as cerebral infarction, caused by chimney grafts might be possible.

Creation of a bypass using an artificial blood vessel between the left common carotid artery and an artificial blood vessel from the right common carotid artery might have allowed blood flow maintenance. The revascularization of the left subclavian artery is subject to debate, and some institutions consider revascularization unnecessary. However, in all cases in our facility, if the procedure in elective surgery is accompanied by a debranching of the left subclavian artery, we create a bypass from the right subclavian artery into the left subclavian artery using an artificial blood vessel. In the present case, we considered that it would be most advantageous for the patient to decrease the surgical duration as much as possible and to have him transported to the ICU for systemic management as soon as the bleeding was under control; therefore, revascularization of the left subclavian artery was not performed. However, physical findings revealed that an impairment of blood flow had developed in the left upper extremity while the patient was in the ICU; therefore, the creation of a right subclavian artery-left subclavian artery bypass using an artificial blood vessel was subsequently performed. Observations conducted at the time of the anastomosis between the artificial blood vessel and the left subclavian artery showed a dissociation of the left subclavian artery, which may have caused the impairment of blood flow.

In this case, we did not administer antiplatelet drugs to the patient. Usually, we give the patients the drug to prevent stroke associated with chimney graft or carotid artery bypass, but this patient was 95 years old. We thought that the risk of bleeding complication was bigger than benefit of antiplatelets.

In the treatment of aortic arch aneurysm ruptures, there is a need to evaluate surgery-related risks preoperatively and to perform surgery as early as possible to stabilize hemodynamics. In patients for whom artificial blood vessel replacement under thoracotomy may pose high risks, stent grafting using the chimney graft technique could be an effective treatment option. However, the long-term prognosis has not been examined and remains to be determined in the future.
